# Is It Time to Move Beyond Visual Inspection With Acetic Acid for Cervical Cancer Screening?

**DOI:** 10.9745/GHSP-D-18-00206

**Published:** 2018-06-27

**Authors:** Shannon L. Silkensen, Mark Schiffman, Vikrant Sahasrabuddhe, John S. Flanigan

**Affiliations:** a Center for Global Health, National Cancer Institute, National Institutes of Health, Bethesda, MD, USA.; b Division of Cancer Epidemiology and Genetics, National Cancer Institute, National Institutes of Health, Bethesda, MD, USA.; c Division of Cancer Prevention, National Cancer Institute, National Institutes of Health, Bethesda, MD, USA.

## Abstract

Newly emerging low-cost molecular assays and improved visual tests for cervical cancer screening call into question the role of visual inspection with acetic acid (VIA). VIA-based screening continues to offer a low-cost, single-visit approach for screening. However, VIA is highly rater-dependent and has problematic accuracy. RNA, DNA, and protein tests are now available. They offer greater accuracy and the option for self-sampling, but the testing kits are expensive. As these new options continue to improve, the time to move beyond VIA is fast approaching.

See related article by Ouedraogo.

In this issue of GHSP, Yacouba Ouedraogo and colleagues describe successes and lessons from a limited scaling up of a cervical cancer prevention program in Burkina Faso based on visual inspection with acetic acid (VIA).^1^ Is now the time to ramp up cervical cancer screening and, if so, should VIA be included? Ouedraogo et al.'s commitment to measuring the impact of the program provides data to examine this question.

## WHAT IS THE BURDEN OF CERVICAL CANCER?

Cervical cancer is highly prevalent in sub-Saharan Africa; the disease can strike women young, prompting the decision to start screening at age 25 in Burkina Faso. The median age for associated mortality is in the early 50s, often during women's most productive years when family and community depend on them.

**FIGURE 1:**
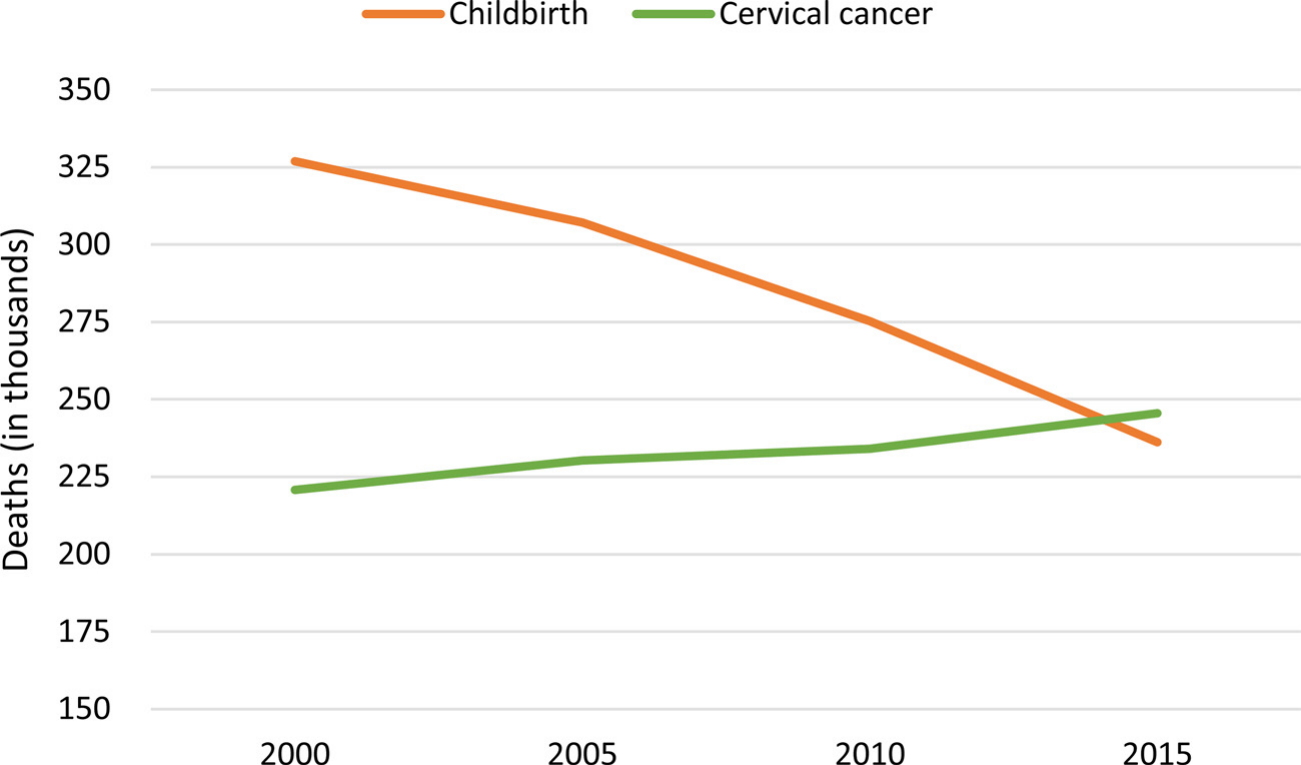
Deaths From Childbirth and Cervical Cancer, 2000–2015 Source: IHME (2016).^2^

Cervical cancer mortality is stubbornly persistent in many low- and middle-income countries. The striking progress seen in decreasing maternal-fetal mortality and infectious disease deaths is not seen for this disease. In fact, the Global Burden of Disease models show that we are at a crossover point with cervical cancer mortality exceeding maternal deaths during childbirth (Figure 1).

We are now at a crossover point with cervical cancer mortality exceeding maternal deaths during childbirth.

## WHAT IS THE ROLE OF PERSISTENT HPV INFECTION IN DEVELOPMENT OF CERVICAL CANCER?

**FIGURE 2:**
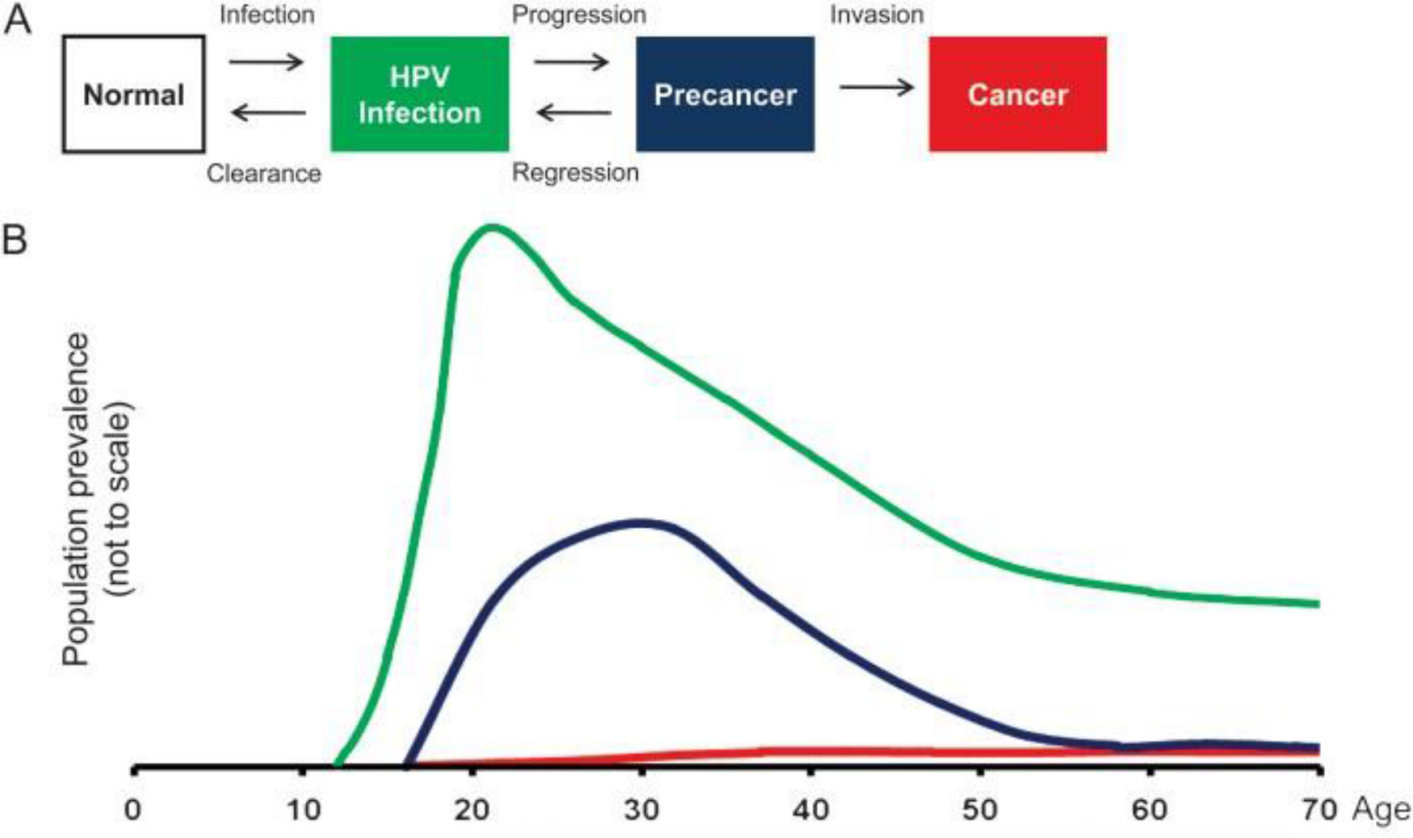
Long-Time Course of Progression From HPV Infection to Cancer Abbreviation: HPV, human papillomavirus. Source: Schiffman (2011).^3^

Human papillomavirus (HPV) is a highly prevalent virus and efficiently transmitted through sexual and skin-to-skin contact. Therefore, promoting abstinence or delay of sexual debut are not effective preventative strategies. Persistence of carcinogenic genotypes of HPV infection leads to virtually all cases of invasive cervical cancer. The long interval between persistence of infection with associated precancers and the development of invasive cancers affords the long-time window for screening and early detection of lesions (Figure 2).

## WHAT STRENGTHS CONTRIBUTED TO THE SCREENING PROGRAM'S EXPERIENCE IN BURKINA FASO?

Ouedraogo and colleagues used a VIA-based detection strategy to reach nearly 14,000 women in Burkina Faso. Of those, 985 (8.9%) screened positive and 649 (65.9%) of those were treated with cryotherapy in a single visit. In addition, 200 women required referral for loop electrosurgical excision procedure (LEEP). Because Ouedraogo and colleagues nested their study within Burkina Faso's health care delivery system, women at community hospitals who needed a more extensive cervical excision procedure were referred to nearby district and teaching hospitals.^1^

The strengths of this cervical cancer screening program include:

1. Organized efforts, offering more efficiencies than sporadic or opportunistic screening
2. Single-visit approach, minimizing loss to follow-up
3. Patient education and outreach, a critical determinant of broader community-level acceptance of prevention programs
4. Health workforce education, vital for success of rater-dependent screening approaches such as VIA

There is growing consensus that these strengths are the basis for building impactful screening programs, but are they enough to recommend the broad uptake of VIA-based screening? Considering the pace achieved, 20 million people live in Burkina Faso; approximately 3.3 million are women ages 25–59 years. Ouedraogo and colleagues required 4 years to cover 14,000 women, underscoring the fact that significant additional resources and manpower commitment will be needed to achieve nationwide coverage. The existing program has achieved a great deal, but one can question whether a quality-assured, practically implemented VIA program of the necessary scale could be established and maintained.

## WHAT CAN BE DONE TO ADDRESS THE SCALING CHALLENGE?

VIA screening followed by treatment (“screen-and-treat”) is currently recommended by the World Health Organization (WHO)^4^ as a cervical cancer screening strategy when more accurate approaches are not available. In our opinion, successful VIA programs, while laudable, will face significant scale-up challenges. VIA has the advantage of being inexpensive with a limited supply-chain burden and results that are apparent at the time of exam. Yet unaided VIA has problematic accuracy and is not reliably reproducible for the identification of precancerous lesions. Additionally, other differences in screened populations such as age, parity, and underlying cervical disease burden affect the positive predictive value of VIA. It is also highly dependent on the skill and judgment of the observer.^5^^–^^7^ For example, in a 2017 study by Raifu and colleagues in the Democratic Republic of the Congo, positivity rates of VIA performed by nurses and physicians differed significantly (36.3% versus 30.2%, respectively).^8^ In contrast, in a large study in India conducted by Shastri and colleagues, the positivity rate of VIA performed by trained high school-level educated public health workers was less than 5%.^9^ Although the utility of VIA in downstaging of invasive cancers in previously unscreened women has been demonstrated in large randomized trials,^9^^,^^10^ its utility for scaling up of screening programs for detecting and treating cervical precancers is limited due to the need for intensive quality assurance efforts and ensuring adequate provider training and re-training.^11^ The strengths and limitations of VIA are illustrated in Table 1.

**TABLE 1.** VIA Strengths and Limitations

VIA Strengths	VIA Limitations
Affordable; low per-capita screening costs	High inter-operator variability
Point-of-care results; treatment or referral decisions can be taken in the same visit	Problematic sensitivity, especially for older women with endocervical lesions
Useful for downstaging of cancers in previously unscreened women	Need for investments in high-intensity quality assurance efforts

Abbreviation: VIA, visual inspection with acetic acid.

Successful VIA programs, while laudable, will likely face significant scale-up challenges.

Given the substantial limitations of VIA, it is important to consider implementing alternative approaches that can overcome its limitations^6^ and permit redirecting resources to reach greater numbers of patients.

## IS IT TIME TO SWITCH TO HPV DNA TESTING AND COMPUTER-ASSISTED VISUAL ASSESSMENT?

There are now at least 5 approved, commercially available HPV tests in the United States,^12^ with more in Europe and many more marketed in Asia. This marketplace is competitive and is starting to improve prices and availability of consumables and testing platforms globally. One test has already received WHO prequalification, an important and necessary step for improving access and bulk purchasing for low-income countries that rely on such multilateral mechanisms for regulatory approvals.^13^ Additional tests are on the near horizon and can further improve on affordability and availability in austere practice settings.

The utility of HPV testing in reducing both incidence and mortality due to cervical cancer has been demonstrated in a large community randomized trial in India.^14^ Several head-to-head comparisons in cross-sectional studies and field demonstration projects in settings as diverse as India,^5^^,^^6^ Uganda,^5^ Zambia,^15^ Tanzania,^16^ South Africa,^17^ Nicaragua,^5^ Brazil,^18^ and Argentina^18^ have shown that HPV testing has better overall test performance characteristics than VIA or cytology (Pap smears). The ability to self-collect specimens is a unique advantage of HPV testing as a screening strategy and can be gainfully employed for expanding the reach of screening programs (Table 2). Testing platforms for HPV are often repurposed from those already utilized for testing for HIV, tuberculosis, and other infectious diseases. One example, the Cepheid GeneXpert HPV cartridge test, can correctly identify women with significant cervical neoplasia 90.8% of the time and provides a point-of-care testing format.^19^ Several of these platforms (e.g., the Cepheid GeneXpert) are already in use in low- and middle-income countries although there might be under-utilization challenges that are setting-specific (M. Bates, Cepheid, personal communication, 2018). Nonetheless, the potential to scale-up HPV testing using such platforms is not yet proven.^19^

**TABLE 2.** HPV Testing Strengths and Limitations

HPV Testing Strengths	HPV Testing Limitations
Point-of-care testing or centralized testing, dependent on testing platform and local needs	Majority of HPV infections (especially in young women) are transientand clinically non-significant
Simplicity and potential scalability of self-collection of samples	Lack of specificity for precancer
Reproducible results; not rater-dependent	Higher per-capita tests costs than VIA
Economical due to longer screening intervals possible for HPV-negative women	

Abbreviations: HPV, human papillomavirus; VIA, visual inspection with acetic acid.

HPV testing has better overall test performance characteristics than VIA or cytology.

We conclude that neither VIA nor the currently priced HPV tests can scale to solve the cervical cancer problem. Screening systems will require alternative approaches that are highly accurate yet cost-effective. The expanding platforms for HPV testing, as well as other emerging screening modalities, especially computer-assisted visual evaluation, will undoubtedly lead to increased options to implement cervical cancer screening programs. Increased usage of self-collected samples will ensure wider coverage. Advanced technologies will also reduce the variable interpretation of subjective clinical exams and limit the chances of over- and under-treatment. It bears mentioning that management of positive cervical cancer screening test results virtually always requires a triage test to prevent over-treatment. While VIA, particularly with low-tech adaptations like digital cervicography, can be used for such triage, novel developments in machine learning/artificial intelligence^20^ and novel imaging techniques^21^ are on the horizon and can dramatically improve on performance of current visual inspection approaches. Additionally, improvements in cut-points for sensitivity and specificity of HPV DNA tests and the development of alternative biomarkers for cervical cancer screening could be other approaches to improve accuracy of protocols relying on primary HPV screening.

Overall, we applaud current high-quality VIA-based programs but believe that the future role for VIA is limited. We are confident that using contemporary, high-quality, reproducible tests will soon provide women, researchers, and clinicians with the accurate screening approaches needed for efficient cervical cancer prevention. As we consider the future, we envision successful cervical cancer screening programs will incorporate modern tests into their current health care systems. The quality of these health care systems informs the likelihood of the patient receiving safe, effective, and timely treatment for their disease. Thus, the platform of VIA programs might survive, but the switch to better screening methods will improve the outcomes for women worldwide.

We believe the future role for VIA is limited and that contemporary, high-quality, reproducible tests will soon provide us with the accurate screening approaches needed for efficient cervical cancer prevention.
